# Norms for an Isometric Muscle Endurance Test

**DOI:** 10.2478/hukin-2014-0011

**Published:** 2014-04-09

**Authors:** Sarah L. Strand, John Hjelm, Todd C. Shoepe, Marie A. Fajardo

**Affiliations:** 1 Loyola Marymount University, Los Angeles, CA.; 2 North Park University, Chicago, IL.

**Keywords:** Fitness, testing, exercise evaluation, muscular endurance

## Abstract

Musculoskeletal performance assessment is critical in the analysis of physical training programs in order to prioritize goals for decreasing injury risk and focusing performance goals. Abdominal endurance as part of this analysis is often assessed with techniques that have validity that has been debated in literature. The purpose of this study was to develop normative sex- and athlete-specific percentiles for a trunk stabilization and muscular endurance by using a prone forearm plank test in college-aged students. A second purpose of this study was to investigate the effect of habitual physical activity and the reason for test termination. There were 471 participants (means ± SE; males: n = 194, age 20.4 ± 0.2 years, body height 179.4 ± 0.5 cm, body mass 81.1 ± 1.2 kg; females: n = 277, age 20.2 ± 0.2 years, body height 165.7 ± 0.4 cm, body mass 63.9 ± 0.7 kg) who performed this test to volitional or technique failure. Males produced significantly higher test durations than females (means ± SD; 124 ± 72 seconds vs. 83 ± 63 seconds) and athletes produced significantly longer test durations than non-athletes (123 ± 69 s vs. 83 ± 63 s) but no interaction effects were seen in the variables of sex and athletic status. The activity level was found to have a threshold of influence (>3 times/week) on abdominal endurance that is dose-specific where greater than 5 times/week showed the greatest influence. The fatigue of the abdominals was the termination reason producing the lowest test duration and there was no sex effect on reason for test termination. These normative percentiles for abdominal endurance suggest that the abdominal plank test can now be used as an alternative to other abdominal assessments in college students, but further investigation is warranted prior to confirmation and generalization to other populations.

## Introduction

The abdominal “core” has become an area of intense scrutiny for researchers, practitioners and exercise participants in recent years. The core is the foundation by which all appendicular movement relies and it includes the ability to dynamically stabilize the spine, hips, pelvis, proximal lower limb, and abdominal structures (Akuthota, 2004;Faries and Greenwood, 2007; Kibler et al., 2006;Putnam, 1993). All musculature traversing or supporting these areas is involved with the core including the transverse abdominis, internal and external obliques, and rectus abdominis for the abdominal muscles. In addition, the latissimus dorsi, pectoralis major, hamstrings, quadriceps, iliopsoas, upper and lower trapezius, hip rotators, and glutei make up the remaining muscles of the core (Kibler et al., 2006; Moraes et al., 2009). The core is an important aspect because it is necessary not only in sports performance but in activities of daily living by gaining stability, improving posture, enhancing balance and proprioception (Bird et al., 2006; Hussain et al., 2007; Richardson and Jull, 1995; Warden et al., 1999). In particular because the core plays a critical role in power transfer to the appendicular skeleton, focusing on the core is an essential part of exercise training. As part of any pre-screening for injury risk or the prescription of any exercise program, muscular performance testing is often included as part of a comprehensive needs analysis. The two common categories of muscular assessment include strength and endurance testing where muscular endurance is defined as the ability to sustain a given level of force production over time while muscular strength is defined as the maximum torque exerted by a muscle or muscle group (Joynt et al., 1993; Lieber, 2002). Moreover, previous research has suggested that muscular endurance is functionally more important to the supportive musculature of the core than muscular strength, so testing should focus on endurance (Knudson, 1999).

Sit-ups and curl-ups have long been prescribed in order to improve strength and due to the desire to assess performance according to specificity, they have also become the main ways to assess abdominal endurance. However, sit-ups and even curl-ups have been shown to perhaps be less indicative of endurance and more indicative of muscular strength or muscular power (Hall et al., 1992). Sit-ups with the feet restrained, in particular require hip flexor activation, which greatly aids the sit-up motion and has been hypothesized to increase the risk of injury because of the movement involved in the motion of a sit-up. There are several concerns with the sit-up in addition to the hip flexor activation alternating patterns of lumbar flexion coupled with hyperlordosis that has been linked with increased pressure on lumbar discs (Baxter et al., 2003; Jette et al., 1984; Juker et al., 1998; Liemohm et al., 1988; Mcgill, 1995). In addition, the administration of sit-up and curl-up tests have been criticized because they require a high degree of administrator training and subjective interpretation of form in order to ensure test validity and reliability (Andersson et al., 1997; Knudson, 1999).

Because of the long-term use of sit-up and curl-up assessments in physical education and fitness training, there is an abundance of data on these techniques available. Therefore, much of the existing literature aimed at assessing muscular endurance or the core has been produced through sit-ups and curl-ups, which have resulted in well-established normative data to rank each individual based on their performance. Unfortunately, as outlined previously, there are number of criticisms and challenges to the validity, reliability, and generalizability of these core assessments. As a result, there has been a search for an abdominal and trunk stabilization exercise that will effectively challenge the muscles while minimizing the hypothesized risk of low-back injury (Childs et al., 2010). The forearm plank test, also referred to as a prone bridge, has been theorized to be more functional because it provides for assessment of endurance during an activity requiring simultaneous activation of the entire anterior muscular chain (Bliss and Teeple, 2005). Plank tests recruit anterior core musculature and challenge the core muscles while specifically targeting the external oblique and lateral stabilizers and increased activity of the anterior musculature has shown improved performance (Aggarwal et al., 2010; Schellenber et al., 2007; Schellenberg et al., 2007). The plank test provides an adequate stimulus for endurance training of the rectus abdominis and external oblique abdominis (Ekstrom et al., 2007). It has been shown that the rectus abdominis and external obliques are important for prevention of injury and improved athletic performance (Nadler et al., 2002; Tsai et al., 2004; Watkins et al., 1996).

Therefore, the purpose of this study was to describe the prone plank test as an alternative assessment of muscular trunk endurance through the creation of percentiles for the purpose of ranking college-aged participants in the establishment of data norms separated by sex and athletic status. We proposed to accomplish this through a test that was less complicated to administer, as well as to increase the construct validity of assessing abdominal endurance, and lessen the risk of lower-back injury.

## Material and Methods

### Participants

Following approval of the Human Subject review boards of Institution North Park University and Loyola Marymount University, a total of 471 participants (males: n = 194, females: n = 277) were recruited. Upon completion of oral and written informed consent, participants were screened for health restrictions identified by the seven-item Physical Activity Readiness Questionnaire (Thomas et al., 1992). Participation was voluntary with no compensation involved. A total of 109 (23% of the sample total) NCAA varsity athletes were included in the analysis compared to 361 who at the time of the study were not affiliated with a varsity sport at their respective institution. Participant descriptive data and anthropometrics are displayed in Table 1. Where there was no difference in mean age, males were significantly taller and heavier than females. When separated by athletic status, the same trends held for age, body height and mass but not by sex where athletes were the same age but heavier and taller on average. The participants also represented a spectrum of activity levels where the following levels were reported: never (1%), rarely (13%), 1–2 times/week (26%), 3–5 times/week (41%), and more than 5 times/week (19%). For those participants that were college athletes, the activity levels included their team practice activities and competition. While it may be assumed that the collegiate athletes were at the higher end of the activity spectrum, some were not in-season at the time of testing and thus, may not have demonstrated peak activity levels. The sample comprised in this study represents a heterogeneous group of young adults affiliated with an institution of higher education including that, which would be expected from large urban areas where these institutions reside and in that the final participant pool demonstrated diversity in ethnicity and activity level.

### Measures

Following a brief technique demonstration, and detailed instructions, participants were tested individually. The test procedures were as follows: the subject assumed the forearm plank position with elbows in contact with the ground, such that the humerus formed a perpendicular line to the horizontal plane, directly beneath the shoulders. The forearms were in neutral position and hands were directly in front of the elbows. The participant assumed a rigid anatomical body position so that only their forearms and toes supported the body. This position is characterized by a phalangeal extension, neutral ankle position, knee and hip extension, and neutral spinal positions.

### Procedures

The participants were instructed to statically hold this position as long as possible and verbal cues were provided to the participant briefly in order to promote form adherence for test validity. When the subject assumed the proper position, the investigator started the stopwatch. The test was terminated when (1) the participant fatigued or voluntarily stopped the test, (2) the participant failed to maintain the proper position, (3) the participant reported ill effects from the test (e.g. headache, dizziness, pain not associated with fatigue, etc.), or (4) the investigator noticed signs indicative of ill effects in the participant from the test. Participants were provided cues during the test as technique faltered away from the accepted position. Tests terminated by the investigator occurred when two consecutive corrective cues given to the participant did not result in an adequate correction in form. At the conclusion of the test, each participant gave their primary subjective reason for discontinuation and the duration time of the test to the nearest tenth of a second was recorded. To keep consistent with other types of fatiguing fitness assessments, each subject only performed the test once.

## Statistical Analysis

Analysis at baseline examined between-group differences in age, body height and mass for sex and athletic status respectively. Potential differences in mean duration of test by sex or athletic status were completed with a 2×5 (sex by physical activity) analysis of variance (ANOVA) whereas a multivariate ANOVA was used to detect the reason for termination for the interaction of sex and athletic status. Activity level effect on test duration was examined with a one-way ANOVA with five levels. Percentile rankings were created for sex and athletic status based on the frequency distribution of the participants’ test duration. A Chi-Square analysis was completed to investigate the observed versus expected reasons for test termination. Pearson product correlations were completed to examine potential relationships between the dependent variable (and thus intended variable of prediction) of test duration and the independent variables of both body height and mass by sex and athletic status. All participant quantitative assessment was analyzed using SASW for Mac version 18.0 (IBM; New York, NY) with a statistical significance set at p < 0.05.

## Results

The grand mean (±SD) of all participants for test duration was 100 ± 63 s. Statistical significance was seen between sexes for the mean duration of the test in seconds where males were shown to have test durations 49% higher than females (124 ± 72 s vs. 83 ± 63 s) and thus normative percentile rankings were generated for males and females separately. The resulting median (50^th^ percentile) was found to be 110 s for males and 72 s for females. Since it would be a common assumption that collegiate athletes had higher activity levels as well as higher strength levels, compared to the non-athlete participants, the data for athletes vs. non-athletes were also assessed separately. There was a significant difference seen in mean duration of the test according to athletic status where athletes were found to have test durations 48% higher than non-athletes (123 ± 69 s vs. 83 ± 63 s) and thus different percentile rankings were generated for each of the categorical definitions of athletic status (varsity athletes versus non-varsity athletes). A value of 104 s was found to be the median score for athletes and 83 s for non-athletes. However, examined together as to potential interactions of sex and athletic status, there was a very high alpha probability value (p = 0.78) that was therefore not deemed to be significant. This null finding for an interaction effect between sex and athletic status did not justify the further separation of normative percentile rankings based on sex and athletic status. Table 2 displays the four resulting percentile rankings of the participants in the study with separation by sex and again by athletic status.

**Figure 1.**
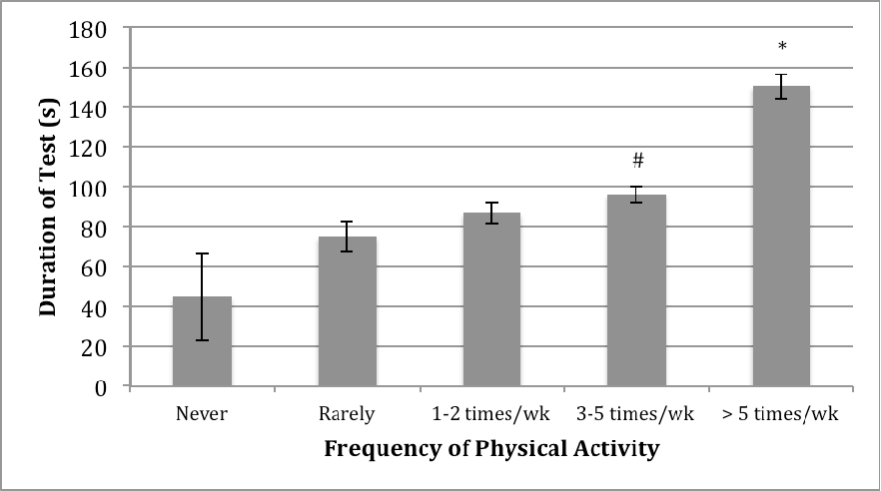
Activity levels of all participants - All values are given as means ± SE. ^*^indicates statistically greater than all other conditions. ^#^ indicates statistically greater than “Never” and “Rarely”. No differences were seen for an interaction effect between sex and activity level resulting in the pooled data shown. Statistical significance was set at p < 0.05.

The effects of activity patterns previous to the testing session on test duration are displayed in Figure 1. There was a positive trend for increased activity to demonstrate longer durations of the plank test. However, statistical significance was seen at only the highest two activity levels. More specifically, those stating that they had been participating in activity patterns more frequently than five times per week had significantly longer test durations than all other conditions. Furthermore, participants with reported activity levels of three-to-five times per week produced longer test durations than those reporting the lowest two activity categories of “none” and “rarely” only.

In order of decreasing frequency for all test participants, the reason for test termination was as follows: legs (n = 151, 32%), arms (n = 150, 32%), abdominals (n = 98, 21%), back (n = 36, 6%), posture (n = 28, 2%), and other (n = 8, 8%). This data included in and resulting from the Chi-square analysis for potential differences in reasons between the sexes are shown in Table 4. There was no statistical difference seen in any reason category between the sexes in the primary reason given for volitional test termination. The resulting totals presented previously were thus pooled accordingly. Arithmetically, abdominal failure or discomfort was the reason for test termination that resulted in the lowest mean test duration followed by the legs, posture, arms, and back. However, of these, only three variables were significantly different. Arm discomfort or failure was the reason associated with the longest test duration compared to abdominals or the legs while the back was significantly longer than abdominals alone. Despite mean difference trends there were no additional differences seen between independent variables for test duration means.

Correlational analysis investigating relationships between body mass and test duration yielded statistically small, negative relationships in all test groups including female non-varsity (r = −0.234; p < 0.001), female varsity (r = −0.321; p = 0.012), male non-varsity (r = −0.318; p < 0.001), and male varsity (r = −0.310; p = 0.009). Correlational analysis investigating relationships between body height and test duration yielded statistically small, negative relationships in all test groups with the exception of female varsity athletes (r = 0.026; p = 0.428). This negative relationship included female non-varsity (r = −0.135; p < 0.05), male non-varsity (r = −0.220; p = 0.005), and male varsity (r = −0.239; p = 0.009).

## Discussion

The primary purpose of this research was to develop normative data for assessing abdominal endurance with a novel, testing protocol hypothetically advantageous to existing methods of abdominal endurance assessment. In this study, 471 college-aged, healthy, males and females representing a diverse ethnic group of urban participants were examined to produce normative data on abdominal endurance. With females demonstrating a statistically lower time to fatigue than males, percentile rankings for males and females were produced separately. Also, because there was a significantly lower time to fatigue for non-athletes as compared to athletes, these percentile rankings were also produced separately (Table 2). Although there were no significant differences seen for mean time to fatigue when evaluated by sex and athletic status, negative relationships in body size in both sex and athletic status justified the creation of percentile norms for these additional four groups: female non-athletes, female athletes, male non-athletes, and male athletes (Table 3).

Previous research discusses the use of situp tests and curl-up tests as the most commonly used ways to assess abdominal endurance. Despite the widespread use of these tests, it has been suggested that there are concerns with these tests both in the objectivity of the tester as well as in the validity of the role of the participant in the assessment. As the pioneer in abdominal testing, sit-up tests in particular have been hypothesized to induce low back pain likely as a result of elevated compressive forces as well as an increase in hip flexor activity especially when the participant became fatigued (Andersson et al., 1997; Baxter et al., 2003; Childs et al., 2010; Diener et al., 1995; Jette et al., 1984; Juker et al., 1998; Liemohm et al., 1988; Mcgill, 1995; Nachemson and Elfstrom, 1970). Second to the sit-up test, more recently research has developed curl-up tests that possibly decrease lumbar spine stress and hip flexor activity that were seen with sit-up tests in the evaluation of abdominal endurance (Juker et al., 1998; Knudson and Johnston, 1995; Nordin and Frankel, 2001; Sternlicht and Rugg, 2003). Resulting work reported that curl-ups were not only able to reduce the stresses on the lumbar spine and decrease hip flexor activity, but were able to reproduce similar abdominal muscle activity to that of sit-ups, creating a safer method to test abdominal endurance (Escamilla et al., 2006). Despite some positive evidence preferring curl-ups to sit-ups, there remains speculation that the development of other methods is warranted (Knudson and Johnston, 1995).

One alternative proposed method to sit-ups and curl-ups in recent years has been the horizontal plank test which has been suggested to have promise in being a more accurate assessment (Schellenberg et al., 2007). The mechanism for this improved efficacy is because the testing is much simpler to administer, as there are fewer directions and increased tester objectivity in the ability to define proper and improper technique. An example of this is in the difference between static and dynamic tests. Where the curl-up and sit-up tests are dynamic assessments requiring movement and a subjective determination of the appropriateness of every repetition, the plank test is initiated with a confirmed starting position and test failure is determined in part when technique sufficiently deviates from this established norm. It could be argued therefore that fewer subjective determinations need to be made in the plank test, which promotes greater validity of the assessment.

Males were found to have significantly longer test durations on the plank test than a corresponding group of females. These data support previous work where sex differences were seen with the plank position (Schellenberg et al., 2007). Examining the possibility that physical activity was a factor in differences for sex for the variable of test duration revealed that arithmetic differences were seen for all levels of physical activity (e.g. never, rarely, 1–2 times/week, etc.). However, likely as a result of large variance in the groups, none of these relationships approached statistical significance. Although the correlation coefficients were small revealing weak relationships, we did find significantly negative correlations between body height and test duration in every group (male varsity, male non-varsity, female nonvarsity) with the exception of female varsity athletes. There were also similar weak relationships between body mass and test duration for every group. These findings support the possibility that body height, mass, and sex are determining factors in predicting time to fatigue. Additionally, noting the relationship between body size and lower test durations, because both athletes and males were on average taller and heavier than non-athletes and females respectively, separate percentiles for athletic status by gender are appropriate.

As expected, both physical activity and athletic status were significantly related to test duration where it was found that with increasing physical activity levels would produce increases in muscular endurance of the abdominal core (Figure 1). Again, a between-groups sex-specific difference was not found in the within-groups categories of activity level or across athletic status. One interesting finding was seen in the dose-response relationship of physical activity and test duration. With all physical activity levels there was an increase in mean test duration time but this only reached a level of significance at higher weekly activity levels. As compared to those that were never or rarely physically active, only statistically higher test durations were seen in the groups active more than three times per week. This suggests a threshold of activity that is necessary to increase abdominal endurance where being active less than three times per week would not be expected to significantly increase fatigue onset. Secondly, the highest activity levels are significantly related to the longest test durations where those participants reporting activity more than five days per week had test durations averaging 150 seconds which was 56% longer than those active 3–5 times per week. The concluding suggestion is that for expected improvements to occur in abdominal endurance, at least three times per week would need to be prescribed and programming over five days per week would be expected to produce the most benefit.

Reason for test termination was found to be significantly related to test duration but no differences were seen between the sexes. Interestingly, abdominals, posture, legs, and other were all statistically similar whereas the back and arms were both significantly higher than the abdominal groups. One explanation for this might be that if the abdominals were undertrained, they would be the weak-link in the abdominal plank and therefore produce discomfort due to fatigue earlier than if they were adequately trained. Adequately trained abdominals would result in participants reporting other muscular regions as being the primary reason for test termination. Shorter tests therefore would be most likely to be terminated because of abdominal discomfort and therefore serve as a valuable diagnostic in determination of priority where additional abdominal training might be prescribed to decrease the chances of injury of the core. An example of this is that our participants citing abdominal discomfort as a primary reason for termination displayed a mean of 87 s with a standard deviation of 49 s. This is in accordance with previous research suggesting athletes should maintain plank positions for at least 60 s (Bliss and Teeple, 2005). This finding supports the efficacy of the plank test for abdominal endurance assessment by producing short test durations and thus low percentile rankings as a result of failure of the abdominals.

The investigation into anthropometrics was included in the hypothesis that higher body mass and height might produce a greater challenge to plank success because greater torque would need to be generated and sustained over time in order to maintain static position resulting from these variables. This was confirmed as statistical differences were seen in mean time to fatigue by sex and separately by athletic status. However, this effect was lost when examined by sex and athletic status concurrently. Males compared to females and athletes compared to non-athletes in this study were both statistically taller and heavier, which suggests body size differences could reduce the observed magnitude of real differences in time to fatigue when compared to females or non-athletes. Future work specifically examining the interaction effects of body size, athletic status, and sex is suggested to elucidate the relative contributions of each to time and fatigue in the forearm plank test

Our data reflect an attempt to produce valid norms for abdominal core endurance in college-aged sex and athletic status. Future work should expand the age of these normative percentile rankings to encompass different age groups which include children, adolescents, as well as a range of adult groups. Secondly, because of training specificity with regard to muscular activity, energetics, and the nature of the kinetic chain, greater attention should be paid to different athletic groups. Third, the physical activity data presented herein were retrospective and thus cross-sectional in nature. This leaves a need for an intervention investigation into the prospective effects of physical activity and core-specific training in the ability to influence abdominal endurance scores as produced with plank tests. A final, albeit more complicated but much more crucial area of future work should investigate the link between abdominal endurance and prospective injury incidence.

## Figures and Tables

**Table 1:** Anthropometric participant data

	**Age (years)**	**Body height (cm)**	**Body mass (kg)**
	
**Total (n=471)**	20.4 ± 0.1	171.0 ± 0.6	70.2 ± 0.8
By Sex			
male (n=192)	20.4 ± 0.2	179.4 ± 0.5^*^	81.1 ± 1.2^*^
female (n=273)	20.2 ± 0.2	165.7 ± 0.4	63.9 ± 0.7
By Athletic Status			
varsity (n=109)	19.9 ± 0.2	174.4 ± 0.9^#^	75.9 ± 1.6^#^
non-varsity (n=361)	20.6 ± 0.2	170.4 ± 0.5	69.3 ± 0.9

All values are given as means ± SE.

* indicates statistically greater than female.

# indicates statistically greater than non-varsity.

Statistical significance was set at p < 0.05.

**Table 2:** Percentiles scores by sex and percentile scores by sport status

	**Time to Fatigue in the Plank-Test (all values in seconds)**			

**Percentile**	**Male (n = 194)**	**Female (n = 275)**	**Non-Varsity (n = 109)**	**Varsity (n = 361)**
**10^th^**	62	35	37	59
**20^th^**	79	48	53	66
**30^th^**	89	58	62	82
**40^th^**	97	63	71	92
**50^th^**	110	72	83	104
**60^th^**	122	84	94	123
**70^th^**	137	95	106	149
**80^th^**	157	108	123	178
**90^th^**	201	142	151	200

**Table 3:** Percentiles score by sex and sport status

	**Time to Fatigue in the Plank-Test (all values in seconds)**			

**Percentile**	**Female Non-Varsity (n = 227)**	**Female Varsity (n = 50)**	**Male Non-Varsity (n = 134)**	**Male Varsity (n = 59)**
**10^th^**	34	45	49	74
**20^th^**	47	59	72	84
**30^th^**	56	63	83	94
**40^th^**	62	74	95	117
**50^th^**	70	87	103	125
**60^th^**	79	97	115	140
**70^th^**	91	110	125	157
**80^th^**	103	162	142	183
**90^th^**	130	194	189	228

**Table 4:** Chi-square results for sex by reason for termination

	**Reason for Test Termination**						
	**Leg**	**Arm**	**Ab**	**Back**	**Posture**	**Other**	**Total**
**Male**							
Frequency	58	60	44	14	4	14	194
% in sex	29.9%	30.9%	22.9%	7.2%	2.1%	7.2%	100%
**Female**							
Frequency	93	90	54	14	4	22	277
% in sex	33.6%	32.5%	19.5%	5.1%	1.4%	7.9%	100%
**Total**							
Frequency	150	151	98	28	8	36	471
Percent	32.1%	31.8%	20.8%	5.9%	1.7%	7.6%	100%

No differences were seen for the expected frequency of termination reason across sex (p < 0.05) for any reason.
